# Information Quality and Semantic Stability of AI Chatbot Responses to Complete Denture Questions: A Turkish–English Benchmarking Study

**DOI:** 10.3390/healthcare14172797

**Published:** 2026-09-01

**Authors:** Sinan Coşkun, Fatma Nur Karaman, Hüseyin Ardıl Uytun, Gülcan Coşkun Akar

**Affiliations:** Department of Prosthodontics, Faculty of Dentistry, Ege University, Bornova 35040, İzmir, Türkiye; fatma.nur.karaman@ege.edu.tr (F.N.K.); huseyin.ardil.uytun@ege.edu.tr (H.A.U.); gulcan.coskun.akar@ege.edu.tr (G.C.A.)

**Keywords:** artificial intelligence, chatbot, complete denture, patient education, large language model, semantic similarity, prosthodontics, health information, digital health

## Abstract

**Highlights:**

**What are the main findings?**

- Expert-rated information quality and embedding-based semantic stability differed among the seven evaluated AI chatbot systems, with GPT-4o showing the numerically highest overall GQS, and Grok the highest semantic stability mean.
- English responses showed higher overall GQS and embedding-based similarity values than Turkish responses, while semantic stability varied across scheduled query sessions and semantically equivalent Turkish prompt formulations.

**What are the implications of the main findings?**

- Chatbot performance should be evaluated separately for information quality, semantic stability, language, and prompt formulation rather than inferred from a single performance metric or model.
- Observed cross-language and repeated-run differences warrant language-specific validation and cautious interpretation; these findings do not establish clinical accuracy, safety, or patient education effectiveness.

**Abstract:**

**Background/Objectives**: Artificial intelligence (AI)-based chatbots are increasingly used as sources of health information, yet the quality and semantic stability of their responses may vary across systems, languages, repeated queries, and prompt formulations. This study compared expert-rated general information quality and embedding-based semantic stability of responses generated by seven AI chatbot systems to expert-derived, patient-oriented complete denture questions in Turkish and English. **Methods**: Twenty-two complete denture-related questions were submitted to seven chatbot systems on three study days across scheduled morning, afternoon, and evening sessions. Two additional semantically equivalent Turkish variants were generated for each original question. Five prosthodontists assessed the original Turkish and English responses using the 5-point Global Quality Score (GQS). Semantic stability was quantified using the multilingual SentenceTransformer checkpoint paraphrase-multilingual-MiniLM-L12-v2 and cosine similarity. To incorporate complete responses, responses were divided into non-overlapping token-based chunks, chunk embeddings were combined into one normalized full-response representation, and the 36 pairwise similarities from the nine repeated responses were averaged to one stability estimate per question–model–language/variant condition. Linear mixed-effects models with question-level clustering were used, with Bonferroni adjustment and partial eta squared effect sizes with 95% confidence intervals. **Results**: AI model significantly affected embedding-based semantic stability (*p* < 0.001; ηp^2^ = 0.820, 95% CI 0.795–0.837) and GQS (*p* < 0.001; ηp^2^ = 0.249, 95% CI 0.152–0.316). Grok had the numerically highest overall semantic stability mean (0.952 ± 0.015), whereas GPT-4o had the numerically highest overall GQS (4.48 ± 0.85). English responses showed higher overall GQS than the original Turkish responses (3.96 ± 0.94 vs. 3.66 ± 1.23; *p* = 0.004) and higher embedding-based similarity than the Turkish conditions overall (*p* < 0.001). No significant overall effect of scheduled study day was observed (*p* = 0.504; ηp^2^ = 0.001), whereas semantic stability differed across scheduled query sessions (*p* < 0.001; ηp^2^ = 0.055), with means of 0.863 ± 0.106, 0.874 ± 0.092, and 0.841 ± 0.140 for morning, afternoon, and evening sessions, respectively. Inter-rater reliability was moderate for English GQS ratings (ICC = 0.675, 95% CI 0.581–0.752) and good for Turkish ratings (ICC = 0.771, 95% CI 0.687–0.833). **Conclusions**: The evaluated chatbot systems differed in expert-rated information quality and full-response embedding-based semantic stability. English responses showed higher overall values than Turkish responses, although cross-language measurement effects of the embedding model cannot be excluded. Differences across scheduled query sessions should be interpreted as run-to-run output variability rather than intrinsic temporal behavior. These findings characterize comparative chatbot performance under the tested conditions but do not establish clinical accuracy, safety, or patient education effectiveness.

## 1. Introduction

The global population is aging rapidly, and the proportion of adults aged 60 years or older continues to increase [1]. Although the prevalence of complete edentulism has declined proportionally in many settings, population aging means that complete dentures and other removable prostheses remain clinically relevant [2]. Older adults using complete dentures may face challenges related to adaptation, hygiene, maintenance, and recognition of situations that require professional review. Inadequate denture hygiene may contribute to biofilm accumulation, denture stomatitis, and, in vulnerable individuals, respiratory complications, such as aspiration pneumonia [3,4]. Adequate patient education is therefore important for appropriate denture use and maintenance, effective hygiene practices, and recognition of complications that may require professional assessment. Previous research has also indicated gaps in denture-related knowledge and hygiene practices among removable denture wearers [5].

Access to oral-health services may also be more difficult for some older adults because of reduced mobility, transportation difficulties, reliance on caregivers, inaccessible facilities, and limited availability of domiciliary services [6,7]. Such barriers may increase the importance of complementary sources of oral-health information, particularly when immediate face-to-face consultation is difficult. Digital approaches, including teledentistry, web-based educational resources, mobile health tools, and conversational artificial intelligence (AI) systems, may therefore supplement conventional information pathways when face-to-face access is difficult, although they do not replace clinical examination or individualized professional advice [8,9,10]. At the same time, increasing use of digital health resources among older populations highlights the need to consider not only accessibility but also the quality, clarity, and reliability of the information provided [8,9].

AI-based chatbots process natural language input and generate human-like responses, and many contemporary systems are based on large language models trained on extensive text corpora [11]. Their potential advantages include rapid access to information, conversational interaction, and the ability to respond in multiple languages. These characteristics may make them particularly attractive for patient-oriented health information, where users may seek immediate explanations about symptoms, self-care, treatment procedures, or device maintenance. However, their use in healthcare also raises important concerns regarding accuracy, safety, completeness, comprehensibility, source transparency, bias, and consistency of generated information [12,13,14]. Large language models may generate plausible but inaccurate or incomplete responses, and the stochastic nature of generative systems means that repeated queries can produce different outputs even when the underlying question remains unchanged [12,13]. For patient education, such variability is relevant because inconsistent information may affect how users interpret and act upon health-related guidance.

Multilingual performance constitutes an additional concern. The amount, diversity, and quality of training data are not uniform across languages, and prior studies have reported differences between English and non-English performance in medical tasks [15,16,17,18]. Consequently, information generated for the same clinical topic may differ in quality or semantic stability according to the language in which the question is asked. This issue is particularly relevant for patient-facing applications, since users are likely to interact with conversational AI systems in their native language rather than in English. Furthermore, patients do not necessarily formulate the same concern using identical wording. Semantically equivalent reformulations of a prompt may alter model outputs, making prompt sensitivity another important consideration when evaluating the reliability of chatbot-generated health information [19,20,21].

Previous studies in healthcare and dentistry have evaluated AI chatbot responses using outcomes such as accuracy, information quality, completeness, readability, comprehensibility, and expert-based ratings [22,23,24,25,26,27,28,29,30]. These studies have provided important evidence regarding the potential usefulness and limitations of conversational AI for patient education and clinical information. Nevertheless, comparatively less attention has been given to whether chatbot responses remain semantically stable when identical questions are repeated across separate query sessions, or when the same underlying patient concern is expressed using semantically equivalent formulations. In addition, studies simultaneously comparing multiple chatbot systems across more than one language and repeated scheduled sessions remain limited in prosthodontics. Evaluating these dimensions may be particularly relevant for complete denture education, where patients may repeatedly seek information about adaptation, hygiene, maintenance, complications, and the need for professional care.

It is also important to distinguish between different dimensions of chatbot performance. Expert-rated general information quality and computational measures of semantic similarity assess related but conceptually different characteristics of responses. Semantic stability should not be interpreted as a direct measure of clinical accuracy or safety, because a chatbot may repeatedly provide semantically similar information that is nevertheless clinically incorrect or incomplete. Conversely, variations in wording do not necessarily indicate clinically meaningful inconsistency. Accordingly, the combined assessment of expert-rated information quality and semantic stability may provide a broader characterization of chatbot performance than either approach alone.

The present study therefore aimed to compare expert-rated general information quality and embedding-based semantic stability of responses generated by seven AI chatbot systems for expert-derived, patient-oriented complete denture questions in Turkish and English. Secondary aims were to examine variation across scheduled repeated query sessions and across semantically equivalent Turkish question formulations. The null hypotheses were that expert-rated information quality and embedding-based semantic stability would not differ by chatbot system or language/variant, and that semantic stability would not vary across the scheduled study days or query-session categories.

## 2. Materials and Methods

### 2.1. Study Design and Ethics

This study was designed as a comparative repeated-measures benchmarking study evaluating AI-generated responses to complete denture-related questions. No patient data or identifiable personal health information were used. Ethical approval was obtained from the Ege University Medical Research Ethics Committee (Decision No. 26-3T/18; 5 March 2026). Five volunteer prosthodontists participated as evaluators, and informed consent was obtained from all evaluators. No compensation was provided.

### 2.2. Question Set Development and Linguistic Variants

An initial pool of 30 patient-oriented questions concerning complete denture use, maintenance, adaptation, and situations requiring professional consultation was developed by a prosthodontist with 25 years of clinical experience, drawing on commonly encountered complete denture topics and publicly available resources from the American Dental Association and the Oral Health Foundation [31,32]. Three prosthodontists then reviewed the candidate pool jointly. Questions considered repetitive, substantially overlapping, or suitable for consolidation into one broader item were revised or combined; items focused primarily on treatment costs, clinic/provider preference, or individual clinician recommendations were excluded. Consensus was reached on a final set of 22 expert-derived patient-oriented questions, the English versions of which are presented in Supplementary Table S1. The final question set covered three principal domains: complete denture use and adaptation; cleaning and storage; and problems or complications requiring professional consultation, including issues related to denture retention and movement, speech and eating, denture adhesives, hygiene procedures, discomfort or mucosal injury, poor fit, and denture fracture or damage. The final number of questions was determined by expert-based content refinement rather than formal power analysis, with the aim of maintaining broad content coverage while minimizing redundancy.

The same three prosthodontists jointly generated two additional Turkish linguistic variants for each original question by modifying word choice and sentence structure while preserving the intended clinical meaning. Semantic equivalence was checked during the joint review process, and the original questions and all variants were subsequently reviewed and approved by the prosthodontist with 25 years of clinical experience who had developed the initial question pool. Thus, the Turkish semantic stability dataset contained 66 prompts (22 original questions plus 44 variants).

The original 22 Turkish questions were translated into English for the cross-language comparison. The Turkish and English versions were reviewed for conceptual and semantic equivalence by three specialist clinicians and one health translator independently of the five clinicians who later performed GQS scoring. The emphasis was on preservation of the intended patient question rather than literal word-for-word correspondence. Formal back-translation was not performed. The five GQS evaluators had fulfilled nationally recognized foreign-language proficiency requirements applicable to specialty training in Türkiye and had experience using English-language scientific literature and/or international scientific publication.

### 2.3. Chatbot Systems, Access Routes, and Querying Environment

Seven chatbot systems were evaluated: ChatGPT-3.5, ChatGPT-4o, Gemini Flash 1.5, Grok, Claude Sonnet 4, DeepSeek, and Microsoft Copilot. The characteristics and querying configurations of the evaluated chatbot systems are summarized in Table 1. Data collection was conducted on 16, 19, and 22 June 2025, organized into scheduled morning, afternoon, and evening sessions. These labels denote collection-session categories rather than strict clock-time windows. Documented query times for API-accessed systems are reported in Supplementary Table S7; equivalent automated timestamp logs were not available for browser-accessed systems. Turkish-language queries were conducted by a study researcher physically located in Türkiye using Türkiye local time, whereas English-language queries were conducted by a collaborating operator physically located in the United Kingdom using UK local time.

For API-accessed systems, questions were submitted using custom Python workflows designed to keep prompts independent and avoid prior conversational context. For browser-accessed systems, separate browser/chat sessions were used according to the study protocol. Automated timestamps were recorded in API logs. Browser interfaces did not provide equivalent automated timestamp logs. Query order was not randomized or counterbalanced across systems or scheduled sessions.

### 2.4. Response Preparation, Blinding, and GQS Assessment

Two response versions were retained for different analytical purposes. The original unedited chatbot outputs were used for all SentenceTransformer-based semantic similarity analyses. No emojis, formatting elements, model-identifying statements, words, or sentences were removed before embedding generation.

For blinded GQS assessment only, separate copies were minimally standardized by a single researcher. Standardization was restricted to explicit chatbot/model self-identification, emojis, and non-informational formatting characters such as quotation marks, asterisks, and hyphens when used as presentation or list-formatting marks. Clinical statements, factual information, recommendations, warnings, explanatory content, sentence wording, and grammar were not intentionally rewritten, paraphrased, added, or removed. The standardized responses were assigned neutral identifiers and presented in randomized Word files.

Five prosthodontists, independent of the experts involved in question development, performed the GQS assessment. One of the nine scheduled query runs was selected by lottery, and the Thursday afternoon session was selected. Responses generated during this same session were used for both the original Turkish and English GQS assessments. The lottery was conducted before the GQS evaluation materials were prepared and before any GQS ratings were assigned. The Thursday afternoon session was selected and subsequently applied consistently across all 22 original questions, all chatbot systems, and both languages. Each model–language condition comprised 22 chatbot responses, each rated by five prosthodontists (110 individual ratings); these 110 ratings do not represent 110 independent chatbot responses. The GQS analysis was designed as a single-run expert-rated quality assessment, whereas all nine repeated generations were used for semantic stability analyses. Consequently, the GQS model rankings reflect the responses generated during the lottery-selected Thursday afternoon session rather than the stability of perceived response quality across the nine repeated runs.

Overall information quality was assessed using the 5-point Global Quality Score (GQS) [33]. The anchors used were: 1, very poor quality and not useful for the patient; 2, poor quality with limited information; 3, moderate quality with some useful information but important omissions; 4, good quality, mostly understandable and useful; and 5, excellent quality, comprehensive and highly suitable for patient education. The GQS was used as a global expert judgment of perceived information quality and usefulness and was not intended as a criterion-based assessment of clinical accuracy or safety.

### 2.5. Semantic Similarity Analysis

Semantic similarity was quantified using the pretrained SentenceTransformer checkpoint sentence-transformers/paraphrase-multilingual-MiniLM-L12-v2 [34,35]. The checkpoint has a native maximum sequence length of 128 tokens and uses mean pooling to generate fixed-dimensional sentence embeddings. Because content beyond this native sequence limit does not contribute to embeddings generated with the standard configuration, response length was audited before finalizing the semantic stability analysis. Of the 5544 responses, 2790 (50.3%) exceeded the 128-token limit, with substantial variation across chatbot systems (Supplementary Table S2). Given the frequency and model-dependent distribution of this exceedance, exclusive reliance on native truncated embeddings could differentially underrepresent later portions of longer responses. Therefore, the final primary semantic stability analysis used a full-response chunking approach so that information throughout each response could contribute to the resulting representation. Responses were tokenized without truncation and divided into non-overlapping chunks sized to remain within the model limit after allowance for special tokens (126 content tokens per chunk). Each chunk was embedded separately; chunk embeddings were combined using a token-count-weighted mean, and the resulting full-response vector was L2-normalized to obtain one embedding per chatbot response. Before encoding, responses were converted to strings and leading/trailing whitespace was removed. No additional lowercasing, punctuation removal, stemming, lemmatization, or language-specific lexical preprocessing was applied. Pairwise semantic similarity was calculated using sklearn.metrics.pairwise.cosine_similarity.

The analysis environment comprised Python 3.13.4, sentence-transformers 5.0.0, transformers 4.53.1, scikit-learn 1.7.0, NumPy 2.3.1, PyTorch 2.7.1, and tokenizers 0.21.2. The same full-response chunking and embedding pipeline was applied to all chatbot systems and both languages in the primary analysis. The multilingual checkpoint was trained for multilingual sentence representation and has been used in Turkish semantic similarity research [36]. Subsequent Turkish benchmarking has also reported substantial performance for this checkpoint on Turkish STS data; however, identical calibration or measurement equivalence between Turkish and English cannot be assumed [37]. Detailed response length distributions and the number and proportion of responses exceeding the checkpoint’s native 128-token limit for each AI model and language/variant condition are provided in Supplementary Table S2.

For each question × AI model × language/variant condition, nine repeated responses yielded 36 unique pairwise cosine-similarity values. Because these pairwise values repeatedly reuse the same underlying responses, the 36 values were not treated as independent observations. For the overall semantic stability analysis, the arithmetic mean of all 36 pairwise similarities was calculated separately for each question, producing one stability value for each question × AI model × language/variant condition. Thus, within each model–language/variant condition, Q1–Q22 each contributed one overall stability value to the mixed-effects model. For the scheduled-day analysis, three question-level day-specific values were produced for each question × AI model × language/variant condition: one for Monday, one for Thursday, and one for Sunday. Each day-specific value was the arithmetic mean of the three pairwise similarities among that day’s morning, afternoon, and evening responses. Accordingly, for a given model (e.g., GPT-3.5), Q1–Q22 were represented separately for Monday, Q1–Q22 for Thursday, and Q1–Q22 for Sunday. For the scheduled-session analysis, three question-level session-specific values were produced analogously for morning, afternoon, and evening. Each session-specific value was the arithmetic mean of the three pairwise similarities for that same session category across the three study days.

To assess the sensitivity of the findings to the embedding strategy, the same question-level aggregation and mixed-effects analyses were additionally performed using embeddings generated with the checkpoint’s native 128-token truncation. These native-truncation analyses were treated as sensitivity analyses, whereas the full-response chunked embeddings constituted the primary analysis.

### 2.6. Statistical Analysis

Statistical analyses were performed using IBM SPSS Statistics version 25. Linear mixed-effects models were used to evaluate semantic stability and GQS outcomes while accounting for clustering by question. For the principal semantic stability model, the analytical unit was one aggregated stability estimate per question × AI model × language/variant condition (22 questions × 7 models × 4 language/variant conditions; N = 616). Fixed effects were AI model, language/variant, and their interaction. For the scheduled-day model, the analytical unit was one aggregated stability estimate per question × AI model × language/variant × day condition (N = 1848), with AI model, language/variant, scheduled day, and all corresponding two- and three-way interactions entered as fixed effects. The scheduled-session model used the analogous question × AI model × language/variant × session unit (N = 1848), with AI model, language/variant, scheduled query session, and all corresponding interactions as fixed effects. For the GQS analysis, the five evaluator ratings for each question × model × language condition were summarized using the median, yielding 22 × 7 × 2 = 308 question-level observations; fixed effects were AI model, language, and their interaction. In all mixed models, question number was specified as the subject-level clustering variable, a random intercept for question was included, a variance components covariance structure was used, and parameters were estimated using restricted maximum likelihood (REML). Denominator degrees of freedom for the fixed-effects tests were estimated using the Satterthwaite approximation. Fixed effects were evaluated using Type III ANOVA F tests. Bonferroni adjustment was applied to multiple comparisons. Effect sizes were reported as partial eta squared (ηp^2^), and their 95% confidence intervals were calculated by numerically inverting the noncentral F distribution using the observed F statistics and corresponding degrees of freedom. Inter-rater reliability among the five prosthodontists was assessed using a two-way mixed-effects, absolute-agreement, average-measures intraclass correlation coefficient (ICC), with 95% confidence intervals. Descriptive summaries are presented as mean ± standard deviation, and statistical significance was set at *p* < 0.05.

## 3. Results

### 3.1. Overall Embedding-Based Semantic Stability Across AI Models and Language Variants

As shown in Table 2, the mixed-effects analysis demonstrated significant main effects of AI model (F = 431.109, *p* < 0.001; ηp^2^ = 0.820, 95% CI 0.795–0.837) and language/variant (F = 16.380, *p* < 0.001; ηp^2^ = 0.080, 95% CI 0.039–0.121), together with a significant AI × language/variant interaction (F = 9.205, *p* < 0.001; ηp^2^ = 0.226, 95% CI 0.146–0.260). Grok had the numerically highest overall semantic stability mean (0.952 ± 0.015), followed by GPT-4o (0.928 ± 0.024) and Gemini (0.921 ± 0.026), whereas Copilot and DeepSeek had the lowest overall means (0.702 ± 0.088 and 0.747 ± 0.095, respectively).

Across language conditions, English produced the highest overall embedding-based similarity value (0.879 ± 0.081), followed by TR1 (0.858 ± 0.103), TR2 (0.851 ± 0.123), and TR3 (0.847 ± 0.109). English differed significantly from the Turkish language/variant conditions in the Bonferroni-adjusted comparisons, whereas the absolute differences among the Turkish variants remained comparatively small. Detailed model-specific and language/variant-specific descriptive values and Bonferroni-adjusted comparisons are provided in Supplementary Table S3.

### 3.2. Expert-Rated GQS and Inter-Rater Reliability

The results of the GQS analysis are presented in Table 3. GQS differed significantly according to AI model (F = 15.088, *p* < 0.001; ηp^2^ = 0.249, 95% CI 0.152–0.316) and language (F = 8.623, *p* = 0.004; ηp^2^ = 0.031, 95% CI 0.003–0.081), whereas the AI × language interaction was not statistically significant (F = 1.273, *p* = 0.270; ηp^2^ = 0.027, 95% CI 0–0.053).

GPT-4o had the numerically highest overall GQS (4.48 ± 0.85), followed by Grok (4.30 ± 0.98) and Gemini (4.16 ± 1.18). The overall English GQS mean was 3.96 ± 0.94, compared with 3.66 ± 1.23 for the original Turkish questions. Post hoc comparisons did not consistently demonstrate statistically significant superiority of the numerically leading model over the next-ranked model; therefore, numerical ranking was distinguished from statistical superiority. Detailed model-by-language GQS values and Bonferroni groupings are provided in Supplementary Table S4.

Inter-rater reliability results are summarized in Table 4. Agreement was moderate for English GQS ratings (ICC = 0.675, 95% CI 0.581–0.752) and good for Turkish GQS ratings (ICC = 0.771, 95% CI 0.687–0.833). The overall ICC was 0.738 (95% CI 0.671–0.791). These estimates were obtained using a two-way mixed-effects, absolute-agreement, average-measures ICC model.

### 3.3. Variation Across Scheduled Study Days

The mixed-effects results for scheduled study day are presented in Table 5. There was no significant overall main effect of scheduled study day on semantic stability (F = 0.686, *p* = 0.504; ηp^2^ = 0.001, 95% CI 0–0.005). The overall semantic stability means were 0.858 ± 0.116 on Sunday, 0.855 ± 0.119 on Monday, and 0.858 ± 0.115 on Thursday.

Significant AI × day (F = 5.156, *p* < 0.001), language/variant × day (F = 2.693, *p* = 0.013), and AI × language/variant × day (F = 1.921, *p* = 0.001) interactions were observed. These findings indicate model- and language-specific variation across scheduled repeated runs rather than a general study-day effect. Detailed model × language/variant × day descriptive values and Bonferroni-adjusted groupings are presented in Supplementary Table S5.

### 3.4. Variation Across Scheduled Query Sessions

The results for scheduled query sessions are shown in Table 6. Semantic stability differed significantly across the scheduled morning, afternoon, and evening query sessions (F = 51.018, *p* < 0.001; ηp^2^ = 0.055, 95% CI 0.036–0.077). The corresponding descriptive means were 0.863 ± 0.106 for morning sessions, 0.874 ± 0.092 for afternoon sessions, and 0.841 ± 0.140 for evening sessions.

Significant AI × session (F = 21.135, *p* < 0.001) and AI × language/variant × session (F = 4.182, *p* < 0.001) interactions were also observed, whereas the language/variant × session interaction was not significant (F = 0.751, *p* = 0.609). Because only one stochastic generation was obtained for each question at each scheduled time point, these findings are interpreted as variation across scheduled query runs rather than evidence of intrinsic time-of-day behavior. Detailed model × language/variant × scheduled-session descriptive values and Bonferroni-adjusted groupings are presented in Supplementary Table S6.

### 3.5. Sensitivity Analysis

Sensitivity analyses based on the checkpoint’s native 128-token truncated embeddings were performed using the same question-level aggregation and mixed-effects modeling strategy as the primary full-response chunked analyses. Although the absolute semantic stability estimates and some effect-size estimates differed between embedding strategies, the principal inferential conclusions were preserved. The effects of AI model and language/variant and their interaction remained statistically significant, the overall scheduled-day effect remained non-significant, and the scheduled-session effect remained significant. Detailed results of the sensitivity analyses are provided in Supplementary Table S8.

## 4. Discussion

This comparative repeated-measures benchmarking study demonstrated differences among seven AI chatbot systems in both expert-rated general information quality and embedding-based semantic stability for complete denture-related questions. GPT-4o had the numerically highest overall GQS, whereas Grok had the numerically highest overall semantic stability mean. English responses showed higher overall GQS and embedding-based similarity values than the corresponding Turkish condition. No significant overall main effect of scheduled study day was identified, whereas semantic stability differed across scheduled morning, afternoon, and evening query sessions. In addition, semantically equivalent Turkish question formulations produced small but statistically detectable differences in embedding-based similarity. Taken together, these findings indicate that chatbot outputs vary according to the system used, language, prompt formulation, and repeated query session; however, the outcomes evaluated in this study characterize expert-perceived information quality and semantic stability rather than clinical accuracy, safety, or effectiveness for patient education.

The two principal outcome measures were selected to evaluate complementary aspects of chatbot performance. GQS provides a global expert judgment of overall information quality and perceived usefulness, whereas SentenceTransformer-based cosine similarity quantifies semantic proximity across independently generated responses. These measures should not be considered interchangeable. A chatbot may repeatedly produce semantically similar responses that contain the same clinical error, while two clinically acceptable responses may differ considerably in wording and structure. Similarly, a high GQS does not provide criterion-based confirmation that every statement within a response is clinically accurate or safe. Recent patient education studies have therefore adopted multidimensional assessment frameworks. Koluman et al. evaluated the accuracy and readability of chatbot responses concerning rotator cuff tears; Özer Aslan and Aslan assessed quality, readability, and clinical accuracy in maternal lactation support; and Jung et al. compared the performance of large language model chatbots for radiation therapy education [38,39,40]. Collectively, these studies illustrate that chatbot performance in patient-oriented contexts is multidimensional and may vary according to both the system and clinical domain. Accordingly, the present findings should be interpreted as a comparative assessment of expert-rated information quality and semantic stability under the tested conditions rather than as validation of the chatbots as autonomous patient education tools.

Marked between-model differences were observed for both study outcomes. Grok had the highest overall full-response semantic stability mean, followed by GPT-4o and Gemini, whereas Copilot and DeepSeek showed comparatively lower semantic stability values. GPT-4o retained the numerically highest GQS. Similar between-system variability has been reported in previous healthcare and dental evaluations of large language models [22,23,24,25,26,27,28,29,30]. Such differences are plausible because commercially available chatbot systems may differ in training data, model architecture, alignment procedures, inference settings, system-level instructions, and interface-level routing. Nevertheless, the numerical ranking observed in the present study should not be interpreted as evidence of universal superiority of one chatbot. Post hoc comparisons did not consistently separate the numerically leading systems, and no established minimum clinically important difference exists for either GQS or embedding-based semantic similarity. Therefore, absolute differences, confidence intervals, effect sizes, and post hoc comparison patterns should be considered alongside numerical rankings.

The magnitude of the observed effects provides additional context for these comparisons. For full-response embedding-based semantic stability, the effect associated with AI model was larger than that associated with language/variant, while a significant AI × language/variant interaction was also observed. A similar pattern was observed for GQS, for which the AI-model effect was appreciably larger than the language effect. Thus, although language-related differences were statistically detectable, the identity of the chatbot system represented a more prominent source of variation under the tested conditions. This distinction is important because statistically significant findings should not automatically be interpreted as practically meaningful, particularly when absolute differences are small.

English responses received higher overall expert-rated GQS values than Turkish responses. Differences between English and non-English performance have also been reported in previous medical evaluations of multilingual large language models [15,16,17,18]. Such differences have been observed across clinical question-answering, diagnostic, and educational tasks and may reflect unequal representation of languages in training corpora, language-specific optimization, and differences in model performance across linguistic contexts. The present findings are therefore consistent with the broader literature indicating that chatbot performance should not be assumed to transfer uniformly from English to other languages. From a practical perspective, this emphasizes the importance of evaluating patient-facing AI systems directly in the language in which patients are expected to use them rather than extrapolating solely from English-language performance.

The higher embedding-based similarity observed for English responses requires additional caution. The same multilingual SentenceTransformer checkpoint and full-response chunking pipeline were applied to English and Turkish responses, and the selected model has previously been used in Turkish semantic similarity research [36]. However, use of a common multilingual encoder does not establish equivalent measurement characteristics across languages. Turkish morphology, tokenization, response length, information ordering, and language-specific representation quality may influence cosine similarity values independently of the underlying stability of chatbot responses. Consequently, the lower Turkish similarity values observed in this study cannot be attributed exclusively to poorer chatbot stability. They should instead be interpreted as lower embedding-based semantic similarity under the selected multilingual measurement framework. Although subsequent Turkish benchmarking has demonstrated substantial performance of this checkpoint on Turkish semantic similarity tasks [37], equivalent calibration between Turkish and English has not been established for dental health chatbot responses. Validation against human semantic similarity judgments and analyses using alternative multilingual encoders would therefore strengthen future cross-language comparisons.

The comparison between the native 128-token and full-response chunked analyses also provides insight into the influence of sequence length truncation on the present findings. Overall, 2790 of 5544 responses (50.3%) exceeded the checkpoint’s native sequence limit, and this exceedance was strongly model dependent. Incorporating the complete responses through chunking changed the absolute semantic stability estimates and the magnitude of some model- and language-related effects, indicating that truncation was not analytically negligible. Nevertheless, the principal inferential pattern was preserved: the effects of AI model and language/variant and their interaction remained significant, the overall scheduled-day effect remained non-significant, and the scheduled-session effect remained significant. The full-response chunked analysis was therefore retained as the primary analysis because it allowed the complete response to contribute to the embedding, whereas the native 128-token analysis served as a sensitivity analysis of the robustness of the principal conclusions.

The scheduled repeated-query analyses further demonstrated that chatbot outputs were not completely deterministic. Although the overall main effect of study day was not significant, significant interactions involving AI model, language/variant, and day indicated that individual systems and language conditions varied differently across repeated runs. Variability across repeated administrations of identical or similar prompts is compatible with the stochastic generation mechanisms of large language models. The present findings therefore do not support a general intrinsic “day effect”; rather, they indicate model- and condition-specific variation across repeated querying.

Semantic stability also differed across the scheduled morning, afternoon, and evening query sessions, with the evening condition showing the lowest overall descriptive mean. However, this result should not be interpreted as evidence that querying an AI chatbot in the evening inherently reduces semantic stability. Only one stochastic generation was obtained for each question at each scheduled time point, and the design did not include replicate generations within individual time slots. Therefore, ordinary generation-to-generation variability, query sequence, hidden platform routing, provider-side updates, or other unobserved factors cannot be separated from the scheduled-session effect. The most appropriate interpretation is that output variation was observed across repeated scheduled runs. Future studies using multiple independent generations within each time slot, randomized or counterbalanced query order, and more tightly standardized access conditions would be required to investigate whether any reproducible temporal pattern exists.

The Turkish linguistic variants provide an additional perspective on output stability. Although the overall means of TR1, TR2, and TR3 were relatively close, statistically detectable differences were observed among some formulations and within specific chatbot systems. Previous studies on prompt sensitivity have demonstrated that relatively small changes in wording, syntax, or prompt structure can alter large language model outputs [19,20,21]. The present findings extend this issue to patient-oriented prosthodontic questions designed to preserve the same underlying clinical meaning. In real-world use, patients are unlikely to phrase identical concerns using standardized wording; therefore, sensitivity to semantically equivalent reformulations may influence the reproducibility of chatbot-generated health information. At the same time, the small absolute differences between the Turkish variant means illustrate why statistical significance should not automatically be equated with practical importance.

Inter-rater reliability for GQS was moderate for English responses and good for Turkish responses, with an overall ICC of 0.738. These results indicate a meaningful degree of agreement among the five prosthodontist evaluators while also demonstrating that global quality assessment retains a subjective component. Clinicians may differ in their interpretation of completeness, usefulness, and suitability for patient communication even when standardized GQS anchors are used. Because the ICC was estimated using a two-way mixed-effects, absolute-agreement, average-measures model, these reliability estimates primarily characterize agreement among the specific evaluator panel used in this study and should not automatically be generalized to all prosthodontists.

The study has several strengths. Seven chatbot systems were evaluated simultaneously, providing broader model coverage than studies focusing on a single system or a small number of platforms. The study also incorporated Turkish and English questions, repeated scheduled data collection, and controlled Turkish linguistic variants. The question set was developed through expert-based refinement to cover commonly encountered complete denture topics while reducing redundancy. Five prosthodontists independently evaluated response quality under blinded conditions, while unedited chatbot outputs were retained for computational semantic analysis. Furthermore, mixed-effects modeling was used to address the repeated and clustered structure of the observations, and effect sizes and confidence intervals were reported alongside *p* values, allowing statistical significance to be distinguished more clearly from the magnitude of observed differences.

An additional methodological strength was the explicit evaluation of the encoder’s native sequence length limitation. Response lengths were quantified across all model–language/variant conditions, full-response chunking was used for the primary semantic analysis when frequent and model-dependent native limit exceedance was identified, and the findings were compared with results obtained using native 128-token truncated embeddings. This sensitivity assessment allowed the influence of truncation on effect estimates and the robustness of the principal conclusions to be evaluated directly rather than assumed.

Several limitations should be considered. First, the final set of 22 questions was expert-derived and did not directly involve complete denture wearers during question development. Although the questions were designed to represent commonly encountered patient-oriented topics, they cannot capture the full range of questions encountered in real-world patient interactions. Second, GQS is a global information-quality measure rather than a criterion-based clinical reference standard. Accordingly, the study did not independently establish factual accuracy, clinical safety, completeness of specific recommendations, readability, or actual patient comprehension. Although the evaluators had fulfilled general foreign-language proficiency requirements and had experience using English-language scientific literature and/or international scientific publication, no study-specific assessment was conducted to establish equivalent proficiency in judging patient-facing naturalness, comprehensibility, or usefulness in Turkish and English. Therefore, differences in evaluator language proficiency may have influenced the cross-language GQS comparison. In addition, the GQS assessment was based on responses from a single scheduled run selected by lottery from the nine available runs. Although the same selected run was applied consistently across all questions, chatbot systems, and both languages, the resulting GQS rankings represent that sampled run and do not estimate the stability of perceived response quality across repeated runs. Third, although full-response chunking allowed later portions of long responses to contribute to the semantic representation, the use of non-overlapping chunks and token-count-weighted mean pooling is itself an analytical choice, and combining independently embedded chunks into a single vector may not fully preserve discourse order, cross-chunk dependencies, or long-range semantic relationships. The sensitivity analysis using native truncated embeddings showed that some effect-size estimates changed according to the embedding strategy, although the principal inferential conclusions were preserved. Fourth, although the same multilingual encoder and chunking pipeline were applied to both languages, measurement equivalence between English and Turkish cannot be assumed. Independent human validation of the semantic similarity scores and analysis with an alternative multilingual encoder were not performed. Fifth, the evaluated chatbot systems were accessed through both APIs and browser interfaces. These routes may differ in hidden system instructions, provider-side routing, generation defaults, context handling, and output constraints; consequently, the results characterize the systems as they were practically accessed during the study period rather than isolated foundation models under fully standardized inference conditions. Sixth, Turkish- and English-language querying was performed by different operators in different countries using their respective local time references, introducing an additional protocol difference. Browser-based systems also lacked automated timestamp logs equivalent to those available for API-accessed systems. Finally, only one stochastic generation was obtained for each question at each scheduled time point, limiting the ability to distinguish scheduled-session effects from ordinary run-to-run variability.

Future research should use larger question banks developed with direct patient involvement and combine global expert-rated quality measures with criterion-based assessments of clinical accuracy and safety. Readability, patient comprehension, and the ability of users to recognize inappropriate or potentially harmful advice should also be evaluated. Repeated generations within each scheduled query slot, standardized access routes, randomized query order, and controlled model configurations would permit more precise estimation of within- and between-session variability. Human validation of embedding-based similarity scores and analyses using alternative multilingual encoders would further strengthen cross-language comparisons. Because commercial AI systems are continually updated, longitudinal benchmarking across model versions may also be important for determining whether observed performance patterns remain stable over time.

## 5. Conclusions

The seven evaluated chatbot systems differed in expert-rated general information quality and embedding-based semantic stability. GPT-4o had the numerically highest overall GQS, while Grok had the numerically highest overall semantic stability mean. English responses showed higher overall GQS and embedding-based similarity values than the corresponding Turkish condition; however, language-related measurement effects cannot be excluded. No significant overall study-day effect was observed, while semantic stability varied across scheduled query sessions, a finding that should be interpreted as run-to-run variability rather than intrinsic temporal behavior. Although effect-size estimates changed to some extent when native 128-token truncated embeddings were compared with full-response chunked embeddings, the principal inferential pattern was preserved, supporting the robustness of the main conclusions to the embedding strategy. These results characterize comparative performance under the tested configurations but do not establish clinical accuracy, safety, or patient education effectiveness.

## Figures and Tables

**Table 1:** Characteristics and querying configurations of the evaluated chatbot systems.

System	Identifier/Version Reported	Access Route	Access Dates	Physical Region	Documented Configuration
ChatGPT-3.5	gpt-3.5-turbo	OpenAI API	16, 19, 22 June 2025	Türkiye/UK	Temperature 0.7; no custom system prompt; question text only; independent query; response limit not explicitly set
ChatGPT-4o	gpt-4o	OpenAI API	16, 19, 22 June 2025	Türkiye/UK	Temperature 0.7; no custom system prompt; question text only; independent query; response limit not explicitly set
Gemini Flash 1.5	gemini-1.5-flash-latest	Gemini API	16, 19, 22 June 2025	Türkiye/UK	Provider defaults; no custom system instruction in script; independent generation
Grok	grok-3	xAI API	16, 19, 22 June 2025	Türkiye/UK	Temperature 0.7; max_tokens 1000; web search prohibited by system instruction
DeepSeek	Web interface; backend snapshot not retrospectively verified	Official browser interface	16, 19, 22 June 2025	Türkiye/UK	Authenticated session; DeepThink disabled; interface defaults
Claude	Claude Sonnet 4	Official browser interface	16, 19, 22 June 2025	Türkiye/UK	Standard Sonnet mode; interface defaults
Microsoft Copilot	Quick response mode; underlying model not exposed	Official browser interface	16, 19, 22 June 2025	Türkiye/UK	Quick response mode; interface defaults

For provider aliases and browser interfaces, a more specific backend snapshot was not inferred when it could not be retrospectively verified. Account tier was not consistently documented across all systems and was therefore not included as an analytical factor. API- and browser-based access routes may differ in hidden system instructions, routing, generation defaults, and output constraints.

**Table 2:** Mixed-effects model results and overall embedding-based semantic stability values according to AI model and language/variant.

**Effect**	F *	**Numerator df**	**Denominator df**	***p***	**Partial Eta Squared (** **η** **p^2^), 95% CI**
AI model	431.109	6	567	<0.001	0.820 (0.795–0.837)
Language/variant	16.380	3	567	<0.001	0.080 (0.039–0.121)
AI × language/variant	9.205	18	567	<0.001	0.226 (0.146–0.260)
**AI model**				**Overall semantic stability, mean ± SD**	
Grok				0.952 ± 0.015	
GPT-4o				0.928 ± 0.024	
Gemini				0.921 ± 0.026	
GPT-3.5				0.903 ± 0.035	
Claude				0.857 ± 0.045	
DeepSeek				0.747 ± 0.095	
Copilot				0.702 ± 0.088	

* Linear mixed-effects model. The analytical unit was one question-level aggregate per question × AI model × language/variant condition (N = 616); each aggregate was the arithmetic mean of the 36 unique pairwise cosine similarities among the nine repeated responses. Question was specified as the subject variable with a random intercept, a variance components covariance structure was used, and parameters were estimated using restricted maximum likelihood. Fixed effects were evaluated using Type III ANOVA F tests, with denominator degrees of freedom estimated using the Satterthwaite approximation. Effect sizes are reported as partial eta squared (ηp^2^) with 95% confidence intervals. Bonferroni adjustment was applied to multiple comparisons.

**Table 3:** Mixed-effects model results and descriptive GQS values according to AI model and language.

**Effect**	F *	**Numerator df**	**Denominator df**	***p***	**Partial Eta Squared (** **η** **p^2^), 95% CI**
AI model	15.088	6	273	<0.001	0.249 (0.152–0.316)
Language	8.623	1	273	0.004	0.031 (0.003–0.081)
AI × language	1.273	6	273	0.270	0.027 (0–0.053)
**AI model**		**English GQS**		**Turkish GQS**	**Overall GQS**
Grok		4.45 ± 0.67		4.14 ± 1.21	4.30 ± 0.98
GPT-4o		4.73 ± 0.46		4.23 ± 1.07	4.48 ± 0.85
GPT-3.5		4.18 ± 0.66		3.32 ± 1.32	3.75 ± 1.12
Gemini		4.23 ± 0.92		4.09 ± 1.41	4.16 ± 1.18
Claude		3.59 ± 0.96		3.50 ± 1.01	3.55 ± 0.98
DeepSeek		3.00 ± 0.69		3.05 ± 1.05	3.02 ± 0.88
Copilot		3.55 ± 0.86		3.32 ± 1.04	3.43 ± 0.95

* Both the mixed-effects model and the descriptive GQS values were based on the question-level median of the five evaluator ratings for each question × AI model × language condition (N = 308). Question was specified as the subject variable with a random intercept, a variance components covariance structure was used, and parameters were estimated using restricted maximum likelihood. Fixed effects were evaluated using Type III ANOVA F tests, with denominator degrees of freedom estimated using the Satterthwaite approximation. Effect sizes are reported as partial eta squared (ηp^2^) with 95% confidence intervals. Bonferroni adjustment was applied to multiple comparisons.

**Table 4:** Inter-rater reliability of GQS ratings for English, Turkish, and overall assessments.

Condition	ICC (95% CI)	*p*	Agreement Level
English	0.675 (0.581–0.752)	<0.001	Moderate
Turkish	0.771 (0.687–0.833)	<0.001	Good
Overall	0.738 (0.671–0.791)	<0.001	Moderate

ICC, intraclass correlation coefficient; CI, confidence interval. ICC estimates were calculated using a two-way mixed-effects, absolute-agreement, average-measures model.

**Table 5:** Mixed-effects model results for semantic stability according to AI model, language/variant, and scheduled study day.

Effect	F *	Numerator df	Denominator df	*p*	Partial Eta Squared (ηp^2^), 95% CI
AI model	695.214	6	1743	<0.001	0.705 (0.685–0.722)
Language/variant	22.232	3	1743	<0.001	0.037 (0.020–0.055)
Scheduled day	0.686	2	1743	0.504	0.001 (0–0.005)
AI × language/variant	15.535	18	1743	<0.001	0.138 (0.102–0.159)
AI × day	5.156	12	1743	<0.001	0.034 (0.014–0.046)
Language/variant × day	2.693	6	1743	0.013	0.009 (0–0.017)
AI × language/variant × day	1.921	36	1743	0.001	0.038 (0.006–0.071)

* Linear mixed-effects model. The analytical unit was one day-specific aggregate per question × AI model × language/variant × scheduled day condition (N = 1848); each aggregate was the arithmetic mean of the three pairwise cosine similarities among the morning, afternoon, and evening responses within that scheduled day. Question was specified as the subject variable with a random intercept, a variance components covariance structure was used, and parameters were estimated using restricted maximum likelihood. Fixed effects were evaluated using Type III ANOVA F tests, with denominator degrees of freedom estimated using the Satterthwaite approximation. Effect sizes are reported as partial eta squared (ηp^2^) with 95% confidence intervals. Bonferroni adjustment was applied to multiple comparisons.

**Table 6:** Mixed-effects model results for semantic stability according to AI model, language/variant, and scheduled query session.

Effect	F *	Numerator df	Denominator df	*p*	Partial Eta Squared (ηp^2^), 95% CI
AI model	655.233	6	1743	<0.001	0.693 (0.671–0.710)
Language/variant	24.358	3	1743	<0.001	0.040 (0.023–0.059)
Scheduled query session	51.018	2	1743	<0.001	0.055 (0.036–0.077)
AI × language/variant	13.564	18	1743	<0.001	0.123 (0.087–0.143)
AI × session	21.135	12	1743	<0.001	0.127 (0.094–0.150)
Language/variant × session	0.751	6	1743	0.609	0.003 (0–0.005)
AI × language/variant × session	4.182	36	1743	<0.001	0.080 (0.040–0.085)

* Linear mixed-effects model. The analytical unit was one session-specific aggregate per question × AI model × language/variant × scheduled-session condition (N = 1848); each aggregate was the arithmetic mean of the three pairwise cosine similarities for the same scheduled-session category across the three study days. Question was specified as the subject variable with a random intercept, a variance components covariance structure was used, and parameters were estimated using restricted maximum likelihood. Fixed effects were evaluated using Type III ANOVA F tests, with denominator degrees of freedom estimated using the Satterthwaite approximation. Effect sizes are reported as partial eta squared (ηp^2^) with 95% confidence intervals. Bonferroni adjustment was applied to multiple comparisons.

## Data Availability

The raw prompts and chatbot responses, anonymized rater-level GQS ratings, available API query timestamps, analysis-ready datasets, supplementary statistical tables, token length audit outputs, and analysis scripts supporting the findings of this study are provided in the Supplementary Materials. Automated timestamps were available for API-accessed systems but were not generated by the browser-based interfaces. The standardized response copies and randomized chatbot-order documentation used for the blinded GQS evaluations are also provided.

## Supplementary Materials

The following supporting information can be downloaded at https://www.mdpi.com/article/10.3390/healthcare14172797/s1: Table S1: Patient-oriented denture-related questions; Table S2: Response token-length distributions and frequency of responses exceeding the 128-token encoder limit; Table S3: Detailed model- and language/variant-specific semantic-stability values and Bonferroni-adjusted comparison groupings; Table S4: Detailed GQS descriptive values and Bonferroni-adjusted model comparison groupings; Table S5: Detailed semantic-stability values by AI model, language/variant, and scheduled study day, with Bonferroni-adjusted comparison groupings; Table S6: Detailed semantic-stability values by AI model, language/variant, and scheduled query session, with Bonferroni-adjusted comparison groupings; Table S7: Documented query-session timestamps derived from the raw API response files; Table S8: Sensitivity Analysis Comparing Native 128-Token Truncation and Full-Response Chunking.

## Abbreviations

The following abbreviations are used in this manuscript:

AI	Artificial Intelligence
API	Application Programming Interface
GQS	Global Quality Score
ICC	Intraclass Correlation Coefficient
LMM	Linear Mixed Model
REML	Restricted Maximum Likelihood
