# Supplementary material for: Information Quality and Semantic Stability of AI Chatbot Responses to Complete Denture Questions: A Turkish–English Benchmarking Study
Source: Healthcare (Basel). 2026 Sep 1;14(17):2797. doi: 10.3390/healthcare14172797 (PMC13566250; doi:10.3390/healthcare14172797)
Supplement: Supplementary file 1 [file healthcare-14-02797-s001.zip › Tables_S3-S6_Statistical_Results.pdf]

Supplementary Table S3. *Detailed model- and language/variant-specific semantic-stability values and Bonferroni-adjusted comparison groupings.*

AI Model	Language Variation				Overall
	EN	TR1	TR2	TR3	
Claude	0.888 ± 0.034^{AB}	0.878 ± 0.035^{ABC}	0.828 ± 0.043^{C}	0.833 ± 0.037^{C}	0.857 ± 0.045^{a}
Copilot	0.731 ± 0.081^{D}	0.705 ± 0.078^{DE}	0.678 ± 0.106^{E}	0.693 ± 0.079^{DE}	0.702 ± 0.088^{b}
DeepSeek	0.843 ± 0.064^{AC}	0.743 ± 0.095^{D}	0.697 ± 0.076^{DE}	0.707 ± 0.069^{DE}	0.747 ± 0.095^{c}
Gemini	0.914 ± 0.016^{BFGH}	0.914 ± 0.029^{BFGH}	0.940 ± 0.019^{FGH}	0.917 ± 0.029^{BFGH}	0.921 ± 0.026^{de}
GPT-3.5	0.901 ± 0.036^{BFG}	0.892 ± 0.039^{ABF}	0.918 ± 0.027^{BFGH}	0.899 ± 0.033^{BFG}	0.903 ± 0.035^{d}
GPT-4o	0.929 ± 0.012^{BFGH}	0.918 ± 0.037^{BFGH}	0.942 ± 0.012^{FGH}	0.924 ± 0.023^{BFGH}	0.928 ± 0.024^{e}
Grok	0.948 ± 0.014^{GH}	0.952 ± 0.019^{H}	0.955 ± 0.013^{H}	0.953 ± 0.010^{H}	0.952 ± 0.015^{f}
Overall	0.879 ± 0.081^{a}	0.858 ± 0.103^{b}	0.851 ± 0.123^{b}	0.847 ± 0.109^{b}	

Values are mean ± standard deviation and are based on the primary full-response chunked embeddings. Lowercase letters denote main-effect comparison groupings; uppercase letters denote interaction comparison groupings. Groups sharing at least one letter were not significantly different in the corresponding Bonferroni-adjusted comparisons. EN, English; TR1–TR3, Turkish question formulations.

Across language variants, overall semantic stability differed significantly among AI systems ($p<.001$). Overall means were 0.857 for Claude, 0.702 for Copilot, 0.747 for DeepSeek, 0.921 for Gemini, 0.903 for GPT-3.5, 0.928 for GPT-4o, and 0.952 for Grok. Claude, Copilot, DeepSeek, and Grok each differed from all other systems. Gemini did not differ significantly from GPT-3.5 or GPT-4o, whereas GPT-4o did not differ significantly from Gemini.

Across AI systems, language/variant also had a significant main effect ($p<.001$). The overall mean was highest for English (0.879), followed by TR1 (0.858), TR2 (0.851), and TR3 (0.847). English differed significantly from all three Turkish language/variant conditions, whereas the Turkish variants did not differ significantly from one another.

The AI × language/variant interaction was significant ($p<.001$), indicating model-specific language effects. Within Claude, English differed from TR2 and TR3; within Copilot, English differed from TR2; and within DeepSeek, English differed from TR1, TR2, and TR3. No significant within-model language/variant differences were identified for Gemini, GPT-3.5, GPT-4o, or Grok. Within English, Claude differed from Copilot and Grok, Copilot differed from all other systems, and DeepSeek differed from all systems except Claude. Within TR1, Claude differed from Copilot, DeepSeek, and Grok; Copilot and DeepSeek differed from Gemini, GPT-3.5, GPT-4o, and Grok; and GPT-3.5 differed from Grok. Within TR2, Claude differed from all other systems, and Copilot and DeepSeek each differed from Gemini, GPT-3.5, GPT-4o, and Grok. Within TR3, Claude, Copilot, and DeepSeek showed significant separation from multiple other systems, and GPT-3.5 differed from Grok. These interaction patterns should be interpreted together with the absolute mean differences and the Bonferroni grouping letters shown in the table.

Supplementary Table S4. *Detailed GQS descriptive values and Bonferroni-adjusted model comparison groupings.*

AI Model	English GQS	Turkish GQS	Overall GQS
Claude	3.59 ± 0.96	3.50 ± 1.01	3.55 ± 0.98 [ab]
Copilot	3.55 ± 0.86	3.32 ± 1.04	3.43 ± 0.95 [ab]
Deepseek	3.00 ± 0.69	3.05 ± 1.05	3.02 ± 0.88 [a]
Gemini	4.23 ± 0.92	4.09 ± 1.41	4.16 ± 1.18 [cd]
Gpt3.5	4.18 ± 0.66	3.32 ± 1.32	3.75 ± 1.12 [bc]
Gpt4o	4.73 ± 0.46	4.23 ± 1.07	4.48 ± 0.85 [d]
Grok	4.45 ± 0.67	4.14 ± 1.21	4.30 ± 0.98 [cd]
Overall	3.96 ± 0.94	3.66 ± 1.23	

Values are mean ± standard deviation. Lowercase letters in the Overall GQS column denote Bonferroni-adjusted model comparison groupings; models sharing at least one letter were not significantly different. GQS, Global Quality Score.

Regardless of language, mean GQS differed significantly among AI systems ($p<.001$). Overall GQS means were 3.55 for Claude, 3.43 for Copilot, 3.02 for DeepSeek, 4.16 for Gemini, 3.75 for GPT-3.5, 4.48 for GPT-4o, and 4.30 for Grok. The Bonferroni-adjusted pattern indicated that Claude and Copilot differed from Gemini, GPT-4o, and Grok; DeepSeek differed from Gemini, GPT-3.5, GPT-4o, and Grok; and GPT-3.5 differed from GPT-4o.

Regardless of AI model, English GQS was higher than Turkish GQS (3.96 vs. 3.66; $p=.004$). The AI × language interaction was not statistically significant ($p=.270$), indicating that the magnitude of the language difference did not vary significantly across models in this analysis.

Supplementary Table S5. *Detailed semantic-stability values by AI model, language/variant, and scheduled study day, with Bonferroni-adjusted comparison groupings.*

		Day			
AI Model	Variation	Monday	Thursday	Sunday	Overall
Claude	EN	0.888 ± 0.044^{ABCDEFGHIJK}	0.876 ± 0.057^{ABCDEFGHIJKL}	0.885 ± 0.043^{ABCDEFGHIJKL}	0.883 ± 0.047^{A}
	TR1	0.872 ± 0.042^{ABCDEFGJKL}	0.875 ± 0.047^{ABCDEFGHJKL}	0.894 ± 0.036^{ABCDEFGHIJK}	0.880 ± 0.042^{A}
	TR2	0.843 ± 0.049^{ABCDEFJKLM}	0.842 ± 0.048^{ABCDEJKLM}	0.807 ± 0.076^{LMNO}	0.830 ± 0.061^{B}
	TR3	0.825 ± 0.053^{ADLMN}	0.836 ± 0.056^{ABCDKLMN}	0.829 ± 0.064^{ACDLMN}	0.830 ± 0.057^{B}
	Overall	0.857 ± 0.052^{A}	0.857 ± 0.054^{A}	0.854 ± 0.067^{A}	0.856 ± 0.058^{a}
Copilot	EN	0.643 ± 0.145^{S}	0.785 ± 0.101^{MNOP}	0.758 ± 0.102^{NOPQR}	0.729 ± 0.131^{C}
	TR1	0.689 ± 0.087^{QRST}	0.721 ± 0.078^{PQRST}	0.713 ± 0.127^{PQRST}	0.708 ± 0.099^{CD}
	TR2	0.685 ± 0.136^{QST}	0.703 ± 0.128^{QRST}	0.658 ± 0.120ST	0.682 ± 0.128^{D}
	TR3	0.680 ± 0.106^{QST}	0.720 ± 0.107^{PQRST}	0.736 ± 0.085^{OPQRT}	0.712 ± 0.101^{CD}
	Overall	0.674 ± 0.120^{D}	0.732 ± 0.108^{BC}	0.716 ± 0.114^{BC}	0.707 ± 0.116^{b}
DeepSeek	EN	0.839 ± 0.078^{ABCDJKLM}	0.835 ± 0.088^{ABCDLMN}	0.820 ± 0.081^{ALMN}	0.831 ± 0.082^{B}
	TR1	0.766 ± 0.121^{MNOPR}	0.713 ± 0.134^{PQRST}	0.673 ± 0.121ST	0.717 ± 0.130^{CD}
	TR2	0.690 ± 0.099^{QRST}	0.642 ± 0.112^{S}	0.700 ± 0.120^{QRST}	0.677 ± 0.112^{D}
	TR3	0.709 ± 0.114^{PQRST}	0.660 ± 0.107ST	0.712 ± 0.108^{PQRST}	0.694 ± 0.111^{CD}
	Overall	0.751 ± 0.118^{B}	0.712 ± 0.133^{C}	0.726 ± 0.121^{BC}	0.730 ± 0.125^{c}
Gemini	EN	0.897 ± 0.027^{ABCDEFGHIJK}	0.920 ± 0.019^{EFGHI}	0.920 ± 0.016^{EFGHI}	0.912 ± 0.023^{AEFG}
	TR1	0.912 ± 0.035^{BEFGHIJK}	0.923 ± 0.026^{FGHI}	0.907 ± 0.041^{BCEFGHIJK}	0.914 ± 0.034^{AEFG}
	TR2	0.939 ± 0.024^{GHI}	0.941 ± 0.018^{GHI}	0.937 ± 0.027^{GHI}	0.939 ± 0.023^{EF}
	TR3	0.912 ± 0.037^{BEFGHIJK}	0.915 ± 0.034^{BEFGHIJK}	0.929 ± 0.024^{GHI}	0.918 ± 0.032^{AEFG}
	Overall	0.915 ± 0.034^{EF}	0.925 ± 0.027^{EFG}	0.923 ± 0.030^{EFG}	0.921 ± 0.031^{d}

GPT-3.5	EN	0.901 ± 0.038 [BCDEFGHIJK]	0.904 ± 0.047 [BCDEFGHIJK]	0.906 ± 0.049 [BCEFGHIJK]	0.904 ± 0.044 [AEG]
	TR1	0.889 ± 0.045 [ABCDEFGHIJK]	0.886 ± 0.058 [ABCDEFGHIJKL]	0.893 ± 0.050 [ABCDEFGHIJK]	0.890 ± 0.051 [AG]
	TR2	0.919 ± 0.029 [EFGHIJ]	0.910 ± 0.037 [BEFGHIJK]	0.928 ± 0.036 [GHI]	0.919 ± 0.035 [AEFG]
	TR3	0.910 ± 0.031 [BEFGHIJK]	0.893 ± 0.048 [ABCDEFGHIJK]	0.900 ± 0.039 [ABCDEFGHIJK]	0.901 ± 0.040 [AEG]
	Overall	0.905 ± 0.037 [E]	0.898 ± 0.048 [E]	0.907 ± 0.045 [E]	0.903 ± 0.044 [e]
GPT-4o	EN	0.926 ± 0.018 [GHI]	0.928 ± 0.018 [GHI]	0.929 ± 0.022 [GHI]	0.928 ± 0.019 [EFG]
	TR1	0.925 ± 0.028 [GHI]	0.915 ± 0.042 [EFGHIJK]	0.918 ± 0.046 [EFGHIJ]	0.919 ± 0.039 [AEFG]
	TR2	0.944 ± 0.021 [GHI]	0.939 ± 0.018 [GHI]	0.944 ± 0.013 [GHI]	0.942 ± 0.018 [EF]
	TR3	0.926 ± 0.026 [GHI]	0.923 ± 0.026 [FGHI]	0.929 ± 0.031 [GHI]	0.926 ± 0.028 [EFG]
	Overall	0.930 ± 0.025 [EFG]	0.926 ± 0.029 [EFG]	0.930 ± 0.031 [EFG]	0.929 ± 0.028 [d]
Grok	EN	0.947 ± 0.015 [GHI]	0.950 ± 0.020 [GHI]	0.947 ± 0.018 [GHI]	0.948 ± 0.017 [F]
	TR1	0.949 ± 0.021 [GHI]	0.950 ± 0.034 [GHI]	0.955 ± 0.021 [HI]	0.952 ± 0.026 [F]
	TR2	0.952 ± 0.021 [HI]	0.954 ± 0.021 [HI]	0.955 ± 0.015 [I]	0.954 ± 0.019 [F]
	TR3	0.949 ± 0.017 [GHI]	0.954 ± 0.014 [HI]	0.954 ± 0.012 [HI]	0.952 ± 0.014 [F]
	Overall	0.949 ± 0.018 [FG]	0.952 ± 0.023 [FG]	0.953 ± 0.017 [G]	0.951 ± 0.020 [f]
Overall	EN	0.863 ± 0.116	0.886 ± 0.079	0.881 ± 0.084	0.876 ± 0.095 [a]
	TR1	0.857 ± 0.108	0.855 ± 0.113	0.851 ± 0.126	0.854 ± 0.116 [b]
	TR2	0.853 ± 0.129	0.847 ± 0.136	0.847 ± 0.137	0.849 ± 0.134 [b]
	TR3	0.844 ± 0.121	0.843 ± 0.122	0.856 ± 0.109	0.848 ± 0.117 [b]
	Overall	0.855 ± 0.119	0.858 ± 0.115	0.858 ± 0.116	

Values are mean ± standard deviation and are based on the primary full-response chunked embeddings. Lowercase letters denote main-effect model/language groupings where shown; uppercase letters denote interaction comparison groupings. Groups sharing at least one letter were not significantly different in the corresponding Bonferroni-adjusted comparisons.

Across language variants and study days, semantic stability differed significantly among AI systems ($p<.001$). Overall means were 0.856 for Claude, 0.707 for Copilot, 0.730 for DeepSeek, 0.921 for Gemini, 0.903 for GPT-3.5, 0.929 for GPT-4o, and 0.951 for Grok. Claude, Copilot, DeepSeek, GPT-3.5, and Grok each differed from all other systems, whereas Gemini and GPT-4o did not differ significantly from each other.

Across AI systems and days, language/variant had a significant main effect ($p<.001$): English had the highest mean (0.876), followed by TR1 (0.854), TR2 (0.849), and TR3 (0.848). English differed significantly from all Turkish variants. By contrast, the overall scheduled-day main effect was not significant ($p=.504$), with means of 0.855 on Monday, 0.858 on Thursday, and 0.858 on Sunday.

Several interaction effects were significant. The AI × language/variant interaction was significant ($p<.001$), with the highest model-by-language mean for Grok-TR2 (0.954) and the lowest for DeepSeek-TR2 (0.677). The AI × day interaction was also significant ($p<.001$), with the highest model-by-day mean for Grok on Sunday (0.953) and the lowest for Copilot on Monday (0.674). The language/variant × day interaction was significant ($p=.013$), with English on Thursday showing the highest mean (0.886) and TR3 on Thursday the lowest (0.843); however, Bonferroni-adjusted pairwise comparisons did not identify significant differences among these interaction groups. The three-way AI × language/variant × day interaction was significant ($p=.001$); the highest cell means were observed for Grok-TR1 and Grok-TR2 on Sunday (both 0.955), whereas the lowest was DeepSeek-TR2 on Thursday (0.642). These findings indicate model- and condition-specific variation across repeated scheduled runs rather than a uniform day effect.

Supplementary Table S6. *Detailed semantic-stability values by AI model, language/variant, and scheduled query session, with Bonferroni-adjusted comparison groupings.*

		Session			
AI Model	Variation	Morning	Afternoon	Evening	Overall
Claude	EN	$0.896 \pm 0.046^{ACDEFGHIK}$	$0.891 \pm 0.033^{ACDEFGHIK}$	$0.873 \pm 0.049^{ABCDEFGHIJ}$	0.886 ± 0.044^{ABC}
	TR1	$0.862 \pm 0.046^{ABCDEFGIJL}$	$0.883 \pm 0.047^{ABCDEFGHIK}$	$0.886 \pm 0.045^{ABCDEFGHIK}$	0.877 ± 0.046^{ABD}
	TR2	$0.781 \pm 0.077^{MNOPQRSTU}$	$0.842 \pm 0.038^{ABCDJLMNOP}$	$0.846 \pm 0.050^{ABCDEJLMNO}$	0.823 ± 0.064^{E}
	TR3	$0.827 \pm 0.054^{ABCJLMNOPQ}$	$0.817 \pm 0.037^{ABJLMNOPQR}$	$0.854 \pm 0.051^{ABCDEFIJLM}$	0.833 ± 0.050^{DE}
	Overall	0.841 ± 0.071^{AC}	0.858 ± 0.049^{A}	0.865 ± 0.050^{AB}	0.855 ± 0.058^{a}
Copilot	EN	$0.766 \pm 0.083^{PQRSTUX}$	0.760 ± 0.108^{QRSTUX}	0.651 ± 0.135^{VW}	0.726 ± 0.122^{FG}
	TR1	0.704 ± 0.109^{SVXY}	0.746 ± 0.092^{RSTUXY}	0.691 ± 0.103^{VXY}	0.714 ± 0.103^{FG}
	TR2	0.690 ± 0.136^{VXY}	0.712 ± 0.100^{STVXY}	0.645 ± 0.139^{VWZ}	0.682 ± 0.127^{F}
	TR3	0.679 ± 0.112^{VWY}	0.734 ± 0.082^{STUXY}	0.644 ± 0.116^{VWZ}	0.686 ± 0.110^{F}
	Overall	0.710 ± 0.115^{EF}	0.738 ± 0.096^{E}	0.658 ± 0.123^{D}	0.702 ± 0.116^{b}
DeepSeek	EN	$0.841 \pm 0.072^{ABCDJLMNOP}$	$0.850 \pm 0.086^{ABCDEFJLMN}$	$0.853 \pm 0.066^{ABCDEFIJLM}$	0.848 ± 0.074^{ADE}
	TR1	$0.782 \pm 0.103^{MNOPQRSTU}$	$0.811 \pm 0.110^{BJLMNOPQRU}$	0.673 ± 0.139^{VWY}	0.755 ± 0.131^{G}
	TR2	$0.772 \pm 0.107^{NOPQRSTU}$	$0.798 \pm 0.090^{JLMNOPQRU}$	0.571 ± 0.128^{Z}	0.714 ± 0.148^{FG}
	TR3	$0.770 \pm 0.099^{OPQRSTU}$	$0.785 \pm 0.085^{LMNOPQRTU}$	0.606 ± 0.114^{WZ}	0.720 ± 0.128^{FG}
	Overall	0.791 ± 0.099^{G}	0.811 ± 0.095^{CG}	0.676 ± 0.158^{DF}	0.759 ± 0.134^{c}
Gemini	EN	0.925 ± 0.018^{FGHIK}	0.924 ± 0.020^{EFGHIK}	$0.892 \pm 0.033^{ACDEFGHIK}$	0.913 ± 0.028^{BCHI}
	TR1	$0.915 \pm 0.034^{DEFGHIK}$	0.922 ± 0.037^{EFGHIK}	$0.896 \pm 0.040^{CDEFGHIK}$	0.911 ± 0.038^{BCHI}
	TR2	0.940 ± 0.025^{GHK}	0.944 ± 0.019^{HK}	0.939 ± 0.032^{GHK}	0.941 ± 0.025^{HI}
	TR3	$0.913 \pm 0.037^{DEFGHIK}$	0.924 ± 0.029^{EFGHIK}	$0.912 \pm 0.035^{DEFGHIK}$	0.916 ± 0.034^{BCHI}
	Overall	0.923 ± 0.031^{HI}	0.928 ± 0.028^{HI}	0.910 ± 0.039^{H}	0.921 ± 0.034^{d}

GPT-3.5	EN	$0.898 \pm 0.044^{CDEFGHIK}$	$0.905 \pm 0.050^{CDEFGHIK}$	$0.894 \pm 0.053^{ACDEFGHIK}$	0.899 ± 0.048^{BCH}
	TR1	$0.885 \pm 0.049^{ABCDEFGHIK}$	$0.894 \pm 0.049^{ACDEFGHIK}$	$0.893 \pm 0.041^{ACDEFGHIK}$	0.891 ± 0.046^{ABC}
	TR2	$0.913 \pm 0.040^{DEFGHIK}$	0.920 ± 0.024^{EFGHIK}	$0.915 \pm 0.031^{DEFGHIK}$	0.916 ± 0.032^{BCHI}
	TR3	$0.891 \pm 0.053^{ACDEFGHIK}$	$0.899 \pm 0.034^{CDEFGHIK}$	$0.898 \pm 0.047^{CDEFGHIK}$	0.896 ± 0.045^{BC}
	Overall	0.897 ± 0.047^{BH}	0.905 ± 0.041^{H}	0.900 ± 0.044^{BH}	0.900 ± 0.044^{e}
GPT-4o	EN	0.921 ± 0.021^{EFGHIK}	0.932 ± 0.016^{GHIK}	0.929 ± 0.019^{GHIK}	0.927 ± 0.019^{CHI}
	TR1	0.921 ± 0.030^{EFGHIK}	$0.916 \pm 0.042^{DEFGHIK}$	$0.914 \pm 0.056^{DEFGHIK}$	0.917 ± 0.043^{BCHI}
	TR2	0.946 ± 0.019^{HK}	0.942 ± 0.014^{HK}	0.940 ± 0.016^{GHK}	0.943 ± 0.017^{HI}
	TR3	0.921 ± 0.038^{EFGHIK}	0.921 ± 0.030^{EFGHIK}	0.920 ± 0.034^{EFGHIK}	0.921 ± 0.034^{BCHI}
	Overall	0.927 ± 0.030^{HI}	0.928 ± 0.029^{HI}	0.926 ± 0.036^{HI}	0.927 ± 0.032^{d}
Grok	EN	0.947 ± 0.017^{HK}	0.953 ± 0.016^{K}	0.947 ± 0.018^{HK}	0.949 ± 0.017^{I}
	TR1	0.951 ± 0.021^{K}	0.956 ± 0.016^{K}	0.945 ± 0.030^{HK}	0.951 ± 0.023^{I}
	TR2	0.955 ± 0.017^{K}	0.953 ± 0.017^{K}	0.956 ± 0.016^{K}	0.955 ± 0.017^{I}
	TR3	0.955 ± 0.011^{K}	0.952 ± 0.014^{K}	0.954 ± 0.014^{K}	0.954 ± 0.013^{I}
	Overall	0.952 ± 0.017^{I}	0.953 ± 0.016^{I}	0.951 ± 0.021^{I}	0.952 ± 0.018^{f}
Overall	EN	0.885 ± 0.075	0.888 ± 0.083	0.863 ± 0.112	0.878 ± 0.092^{a}
	TR1	0.860 ± 0.103	0.875 ± 0.092	0.843 ± 0.127	0.859 ± 0.109^{b}
	TR2	0.857 ± 0.123	0.873 ± 0.101	0.830 ± 0.164	0.853 ± 0.133^{bc}
	TR3	0.851 ± 0.112	0.862 ± 0.092	0.827 ± 0.148	0.847 ± 0.120^{c}
	Overall	0.863 ± 0.106^{c}	0.874 ± 0.092^{b}	0.841 ± 0.140^{a}	

Values are mean ± standard deviation and are based on the primary full-response chunked embeddings. Lowercase letters denote main-effect model/session groupings where shown; uppercase letters denote interaction comparison groupings. Groups sharing at least one letter were not significantly different in the corresponding Bonferroni-adjusted comparisons.

Across language variants and scheduled query sessions, semantic stability differed significantly among AI systems ($p<.001$). Overall means were 0.855 for Claude, 0.702 for Copilot, 0.759 for DeepSeek, 0.921 for Gemini, 0.900 for GPT-3.5, 0.927 for GPT-4o, and 0.952 for Grok. Claude, Copilot, DeepSeek, GPT-3.5, and Grok each differed from all other systems, whereas Gemini and GPT-4o did not differ significantly from each other.

Across AI systems and sessions, language/variant had a significant main effect ($p<.001$): English had the highest mean (0.878), followed by TR1 (0.859), TR2 (0.853), and TR3 (0.847). English differed significantly from all Turkish variants, and TR1 also differed significantly from TR3. Scheduled query session had a significant main effect ($p<.001$), with means of 0.863 for morning, 0.874 for afternoon, and 0.841 for evening; all three scheduled-session categories differed significantly in the Bonferroni-adjusted comparisons.

The AI × language/variant interaction was significant ($p<.001$), with the highest model-by-language mean for Grok-TR2 (0.955) and the lowest for Copilot-TR2 (0.682). The AI × session interaction was also significant ($p<.001$), with the highest model-by-session mean for Grok in the afternoon (0.953) and the lowest for Copilot in the evening (0.658). The language/variant × session interaction was not significant ($p=.609$). The three-way AI × language/variant × session interaction was significant ($p<.001$), with the highest cell means for Grok-TR2 in the evening and Grok-TR1 in the afternoon (both 0.956), and the lowest for DeepSeek-TR2 in the evening (0.571). Because the design included one stochastic generation per question at each scheduled time point, these session-related findings should be interpreted as variation across scheduled runs rather than evidence of intrinsic time-of-day behavior.
