# Supplementary material for: Information Quality and Semantic Stability of AI Chatbot Responses to Complete Denture Questions: A Turkish–English Benchmarking Study
Source: Healthcare (Basel). 2026 Sep 1;14(17):2797. doi: 10.3390/healthcare14172797 (PMC13566250; doi:10.3390/healthcare14172797)
Supplement: Supplementary file 1 [file healthcare-14-02797-s001.zip › Table_S1_Questions.pdf]

Supplementary Table S1. *Patient-oriented denture-related questions.*

No	Question
1	Will I remove my dentures every day?
2	Should I take my dentures off when I sleep?
3	What should I do to get used to my dentures?
4	I feel nauseous when I put my dentures on. What should I do?
5	Can I eat everything when I start using my dentures?
6	Is it normal for my dentures to move when I eat or talk?
7	I have difficulty speaking. What can I do to improve my speech with dentures?
8	How should denture adhesives be used, and how frequently should they be renewed?
9	Does consuming coffee or tea, or smoking, harm dentures?
10	How long can I use my dentures, and when should they be replaced?
11	How should dentures be cleaned? Can toothpaste or bleach be used?
12	How should denture cleaning tablets be used?
13	Can a toothbrush be used to clean dentures?
14	I use denture adhesives. How can I remove adhesive residues?
15	How should dentures be stored when they are not in use?
16	When should I consult my dentist regarding my dentures?
17	My dentures feel loose. What should I do?
18	My dentures feel too tight. What should I do?
19	My dentures are causing pain or sore spots. What should I do?
20	A tooth has fallen off my denture. What should I do?
21	My dentures are broken. Can I glue them together?
22	My denture is cracked. What should I do?
