# Supplementary material for: Information Quality and Semantic Stability of AI Chatbot Responses to Complete Denture Questions: A Turkish–English Benchmarking Study
Source: Healthcare (Basel). 2026 Sep 1;14(17):2797. doi: 10.3390/healthcare14172797 (PMC13566250; doi:10.3390/healthcare14172797)
Supplement: Supplementary file 1 [file healthcare-14-02797-s001.zip › Table_S2_Token_Length_Distributions.pdf]

Supplementary Table S2. *Response token-length distributions and frequency of responses exceeding the 128-token encoder limit.*

AI model	Language / variant	n	Tokens, mean ± SD	Median (Q1–Q3)	Min–Max	>128 tokens, n (%)
GPT-3.5	English	198	151.8 ± 72.3	123.5 (95.0–214.0)	33–388	92 (46.5%)
	TR1	198	152.9 ± 62.0	140.5 (97.5–199.3)	67–402	111 (56.1%)
	TR2	198	145.4 ± 62.7	129.5 (94.3–190.8)	50–328	101 (51.0%)
	TR3	198	161.2 ± 62.8	156.0 (104.3–217.8)	53–293	123 (62.1%)
GPT-4o	English	198	341.2 ± 102.1	347.5 (299.3–406.0)	87–582	190 (96.0%)
	TR1	198	278.7 ± 79.3	288.0 (247.0–336.5)	90–432	185 (93.4%)
	TR2	198	297.3 ± 74.0	308.5 (263.5–344.5)	89–432	185 (93.4%)
	TR3	198	272.4 ± 92.7	297.5 (213.0–345.8)	93–443	174 (87.9%)
Gemini Flash 1.5	English	198	423.1 ± 177.7	420.0 (302.8–538.8)	82–766	181 (91.4%)
	TR1	198	384.9 ± 138.0	382.5 (301.0–491.0)	37–678	187 (94.4%)
	TR2	198	409.4 ± 147.2	415.0 (320.3–512.8)	53–730	183 (92.4%)
	TR3	198	391.7 ± 156.8	386.5 (275.3–523.8)	47–686	186 (93.9%)
Claude Sonnet 4	English	198	100.6 ± 33.5	101.5 (67.0–130.8)	47–160	59 (29.8%)
	TR1	198	77.2 ± 14.4	79.0 (66.0–88.0)	41–109	0 (0.0%)
	TR2	198	98.8 ± 35.5	94.0 (69.0–121.0)	48–223	33 (16.7%)
	TR3	198	78.8 ± 28.7	68.5 (55.0–104.3)	35–139	8 (4.0%)
Grok	English	198	572.2 ± 151.9	570.5 (481.8–668.5)	177–980	198 (100%)
	TR1	198	473.6 ± 115.7	466.0 (377.8–543.5)	271–762	198 (100%)
	TR2	198	527.3 ± 170.0	490.5 (410.3–591.3)	264–1284	198 (100%)
	TR3	198	494.7 ± 144.9	468.0 (400.0–559.8)	231–1209	198 (100%)
DeepSeek	English	198	28.0 ± 5.4	28.0 (24.0–31.0)	16–46	0 (0.0%)
	TR1	198	27.6 ± 13.2	24.0 (19.0–32.8)	11–72	0 (0.0%)
	TR2	198	20.8 ± 6.6	20.0 (16.0–24.0)	9–49	0 (0.0%)
	TR3	198	20.7 ± 6.6	20.5 (16.0–24.0)	9–48	0 (0.0%)
Microsoft Copilot	English	198	34.0 ± 8.9	32.0 (27.0–39.0)	20–61	0 (0.0%)
	TR1	198	29.4 ± 7.8	29.5 (24.0–34.0)	15–51	0 (0.0%)
	TR2	198	27.8 ± 7.1	28.0 (22.0–32.8)	13–52	0 (0.0%)
	TR3	198	30.9 ± 10.7	29.0 (24.0–34.8)	15–73	0 (0.0%)

Note: Token counts were obtained using the tokenizer corresponding to the primary SentenceTransformer checkpoint (sentence-transformers/paraphrase-multilingual-MiniLM-L12-v2) without truncation and with special tokens included. The encoder has a maximum sequence length of 128 tokens. Values exceeding 128 therefore indicate responses for which the primary embedding analysis did not incorporate the complete response. Each model–language/variant condition comprised 198 responses (22 questions × 9 scheduled runs). TR1, TR2, and TR3 denote the original Turkish prompt and its two semantically equivalent linguistic variants, respectively.
