# Supplementary material for: Information Quality and Semantic Stability of AI Chatbot Responses to Complete Denture Questions: A Turkish–English Benchmarking Study
Source: Healthcare (Basel). 2026 Sep 1;14(17):2797. doi: 10.3390/healthcare14172797 (PMC13566250; doi:10.3390/healthcare14172797)
Supplement: Supplementary file 1 [file healthcare-14-02797-s001.zip › Table_S7_Query_Timing_and_Session_Map.pdf]

Supplementary Table S7. *Documented query-session timestamps derived from the raw API response files*

Panel A. *Turkish-language query sessions conducted in Türkiye*

Session	Date	Scheduled period	ChatGPT-3.5	ChatGPT-4o	Gemini Flash 1.5	Grok
1	16 Jun 2025	Morning	09:36–09:45	09:15–09:29	09:17–09:21	09:19–09:34
2	16 Jun 2025	Afternoon	14:56–15:06	15:08–15:26	14:57–15:01	14:56–15:11
3	16 Jun 2025	Evening	20:46–20:56	20:57–21:13	20:46–20:50	20:46–21:02
4	19 Jun 2025	Morning	08:55–09:04	09:05–09:22	09:03–09:07	09:01–09:14
5	19 Jun 2025	Afternoon	13:43–13:52	13:53–14:09	13:43–13:48	13:44–13:58
6	19 Jun 2025	Evening	21:07–21:16	20:20–20:36	20:21–20:25	20:22–20:36
7	22 Jun 2025	Morning	09:14–09:23	09:00–09:12	09:00–09:04	09:00–09:13
8	22 Jun 2025	Afternoon	14:20–14:29	14:01–14:19	14:02–14:06	14:07–14:21
9	22 Jun 2025	Evening	20:18–20:27	20:01–20:15	20:02–20:06	20:02–20:17

Panel B. *English-language query sessions conducted in the United Kingdom*

Session	Date	Scheduled period	ChatGPT-3.5	ChatGPT-4o	Gemini Flash 1.5	Grok
1	16 Jun 2025	Morning	10:32–10:35	08:38–08:44	08:39–08:40	08:39–08:42
2	16 Jun 2025	Afternoon	16:36–16:39	16:27–16:32	16:30–16:31	16:28–16:30
3	16 Jun 2025	Evening	20:23–20:26	20:13–20:17	20:06–20:07	20:35–20:38
4	19 Jun 2025	Morning	08:53–08:59	09:00–09:05	08:45–08:46	08:46–08:49
5	19 Jun 2025	Afternoon	15:06–15:09	15:00–15:05	15:01–15:02	15:02–15:05
6	19 Jun 2025	Evening	20:14–20:17	20:07–20:12	20:07–20:08	20:08–20:11
7	22 Jun 2025	Morning	09:17–09:19	09:05–09:10	09:11–09:12	09:06–09:09
8	22 Jun 2025	Afternoon	14:39–14:41	14:08–14:12	14:08–14:09	14:08–14:11
9	22 Jun 2025	Evening	19:40–19:44	19:26–19:30	19:26–19:28	19:26–19:29

Note: Time ranges were derived directly from the timestamp fields in the raw API response files included in the Supplementary Materials. Each Turkish-language API session contained 66 prompts (22 original questions and 44 Turkish linguistic variants), whereas each English-language API session contained 22 prompts. Turkish timestamps are reported in Türkiye local time and English timestamps in United Kingdom local time. Automated timestamp logs were available for ChatGPT-3.5, ChatGPT-4o, Gemini Flash 1.5, and Grok, but not for the browser-accessed Claude Sonnet 4, DeepSeek, and Microsoft Copilot sessions. The labels morning, afternoon, and evening refer to scheduled collection-session categories rather than identical clock-time windows across systems.
