# Supplementary material for: Information Quality and Semantic Stability of AI Chatbot Responses to Complete Denture Questions: A Turkish–English Benchmarking Study
Source: Healthcare (Basel). 2026 Sep 1;14(17):2797. doi: 10.3390/healthcare14172797 (PMC13566250; doi:10.3390/healthcare14172797)
Supplement: Supplementary file 1 [file healthcare-14-02797-s001.zip › Table_S8_128_vs_Chunked.pdf]

Supplementary Table S8. *Sensitivity Analysis Comparing Native 128-Token Truncation and Full-Response Chunking*

Sensitivity analyses were conducted using the same question-level aggregation and mixed-effects modelling framework as the primary analysis. The native 128-token analysis used embeddings generated under the checkpoint's standard sequence-length limit, whereas the primary analysis used full-response chunking with token-count-weighted pooling. All comparisons below therefore reflect the influence of the embedding strategy while preserving the same analytical unit and statistical model structure.

Table S8A. *Comparison of Type III fixed-effects tests under native 128-token truncation and full-response chunking.*

Effect	Embedding strategy	F	Numerator df	Denominator df	p	Partial ηp² (95% CI)
AI model	Native 128-token truncation	295.047	6	567	< 0.001	0.757 (0.725–0.780)
AI model	Full-response chunking	431.109	6	567	< 0.001	0.820 (0.795–0.837)
Language/variant	Native 128-token truncation	23.944	3	567	< 0.001	0.112 (0.065–0.159)
Language/variant	Full-response chunking	16.380	3	567	< 0.001	0.080 (0.039–0.121)
AI model × language/variant	Native 128-token truncation	7.298	18	567	< 0.001	0.188 (0.110–0.219)
AI model × language/variant	Full-response chunking	9.205	18	567	< 0.001	0.226 (0.146–0.260)

Note. The analytical unit was one question-level aggregate per question × AI model × language/variant condition (N = 616). Question was specified as the subject variable with a random intercept; a Variance Components covariance structure and restricted maximum likelihood estimation were used. Fixed effects were evaluated using Type III ANOVA F tests, with denominator degrees of freedom estimated using the Satterthwaite approximation.

Table S8B. *Overall semantic-stability means by AI model under the two embedding strategies.*

AI model	Native 128-token truncation, mean ± SD	Full-response chunking, mean ± SD	Absolute change (chunked − native)
Claude	0.855 ± 0.047	0.857 ± 0.045	+0.002
Copilot	0.702 ± 0.088	0.702 ± 0.088	0.000
DeepSeek	0.747 ± 0.095	0.747 ± 0.095	0.000
Gemini	0.890 ± 0.039	0.921 ± 0.026	+0.031
GPT-3.5	0.890 ± 0.042	0.903 ± 0.035	+0.013
GPT-4o	0.911 ± 0.035	0.928 ± 0.024	+0.017
Grok	0.923 ± 0.036	0.952 ± 0.015	+0.029

Note. Positive values indicate higher mean semantic stability after full-response chunking.

Table S8C. *Overall semantic-stability means by language/variant under the two embedding strategies.*

Language/variant	Native 128-token truncation, mean ± SD	Full-response chunking, mean ± SD	Absolute change (chunked − native)
English	0.873 ± 0.080	0.879 ± 0.081	+0.006
TR1	0.840 ± 0.096	0.858 ± 0.103	+0.018
TR2	0.837 ± 0.115	0.851 ± 0.123	+0.014
TR3	0.831 ± 0.099	0.847 ± 0.109	+0.016

Interpretation of the sensitivity analysis

The native-truncation and full-response chunked analyses yielded the same principal inferential pattern. AI model, language/variant, and the AI model × language/variant interaction remained statistically significant under both embedding strategies. However, the magnitude of the effects changed after incorporating the complete responses. The AI-model effect increased from $\eta_p^2 = 0.757$ to 0.820, the language/variant effect decreased from 0.112 to 0.080, and the interaction effect increased from 0.188 to 0.226.

The descriptive comparison also showed that the influence of chunking was not uniform across chatbot systems. The largest increases in overall semantic-stability means were observed for Gemini (+0.031), Grok (+0.029), GPT-4o (+0.017), and GPT-3.5 (+0.013), whereas Claude changed only minimally (+0.002) and Copilot and DeepSeek were unchanged at the reported precision. This pattern is consistent with the response-length audit showing that exceedance of the checkpoint's native 128-token limit was strongly model-dependent.

Across language/variant conditions, full-response chunking increased the overall mean similarity in all four conditions, with changes ranging from +0.006 for English to +0.018 for TR1. Despite these shifts in absolute values and effect-size estimates, the direction of the main findings was preserved. Accordingly, the native 128-token analysis supports the robustness of the principal inferential conclusions, while the full-response chunked analysis was retained as the primary analysis because it allowed information from the complete chatbot responses to contribute to the embeddings.
